# An assessment of child immunization coverage and its determinants in Sinana District, Southeast Ethiopia

**DOI:** 10.1186/s12887-015-0345-4

**Published:** 2015-04-01

**Authors:** Elias Legesse, Worku Dechasa

**Affiliations:** College of Medical and Health Sciences, Department of Public Health, Wollega University, Nekemte, Ethiopia

**Keywords:** Children, Immunization, Coverage, Vaccination, Determinants, Child mortality and morbidity

## Abstract

**Background:**

Immunization remains one of the most important public health interventions and cost effective strategies to reduce child mortality and morbidity associated with infectious diseases. It is estimated to avert between 2 and 3 million deaths each year worldwide. The objective of this study was to assess complete immunization coverage and its associated factors among children aged 12 to 23 months in Sinana district, Bale Zone, Southeast Ethiopia.

**Methods:**

A cross-sectional community based survey was conducted in Sinana district from December 2012 to January 2013. A modified World Health Organization-Expanded Program on Immunization cluster sampling methods were used for household selection. A total 591 children aged 12 to 23 months and their mothers or caregivers were included in the study. Data were collected by using a pre-tested, interviewer administered questionnaire. Bivariate analysis was employed to identify factors associated with full immunization coverage and multiple logistic regression analysis was performed for those factors that showed statistically significant association in bivariate analysis and investigate independent predictors by controlling for possible confounders and significances of all tests were decided at p-value of 0.05.

**Results:**

More than three fourth (76.8%) of the children aged 12 to 23 months were fully vaccinated by card plus history. Factors significantly associated with full immunization were antenatal care follow up (AOR = 3.7; 95% CI: 2.3, 5.9), being a farmer (AOR = 1.9; 95% CI: 1.1, 3.1), being father with secondary and above educational level (AOR = 3.1; 95% CI: 1.3, 7.4), having household family income greater than 1000 ETB or 52 USD (AOR = 3.2; 95% CI: 1.4, 7.4), those whose average walking time from home to health facilities is less than an hour (AOR = 3.1; 95% CI: 1.5, 6.3), those who had ever discussed about immunization with health extension workers (AOR = 2.4, 95% CI: 1.3, 4.2) and mothers’ with sufficient knowledge on immunization (AOR = 2.5; 95% CI: 1.5, 4.2).

**Conclusions:**

Even though, immunization coverage of children in Sinana district gets improvement over national coverage, yet it is below governmental plan to increase the coverage i.e. 90%. Maternal health care utilization and knowledge of mother about vaccine and vaccine preventable diseases are the main factors associated with complete immunization coverage. It is vital that local programmatic intervention should be strengthened to upgrade awareness of the community on the importance of immunization, antenatal care and working on advancing economic status of community is the way to optimize children’s immunization coverage.

## Background

Universal immunization of children against six preventable diseases (tuberculosis, diphtheria, pertussis, tetanus, polio, and measles) is vital to reduce childhood mortality and morbidity across the world. So, it is one of the indicators of development in most developing countries. The Expanded Program on Immunization (EPI) was launched in 1974 as a global program for controlling and reducing death from Vaccine Preventable Diseases (VPDs). Thus, vaccine coverage is estimated as by convention with Diphtheria, Pertussis and Tetanus-3 (DPT-3) coverage achieved among children aged 12 to 23 months [1,2].

At the end of 2011, immunization was reported to have saved 2 to 3 million lives; nonetheless, in the same year 1.5 million children are estimated to have died (more than 70% live in ten African and Asian countries) from VPDs is a reflection of the incomplete coverage with existing vaccines that persists in many parts of the world. Goal of Global Immunization Vision and Strategy (GIVS) was to reduce global measles deaths by 90% by the year 2010 or earlier [3,4].

The WHO in Africa region and the Global Alliance for Vaccines and Immunization (GAVI) in 2000 have set a goal of reaching ≥80% DPT-3 coverage in every district in greater than 80% of developing countries by 2005. This goal is referred as the “80/80 goal”. To achieve this goal, the GAVI proposed a new approach, Reaching Every District (RED) in 2002 [4].

Millennium Development Goal 4 (MDG-4) aim is a two-thirds reduction of Under-Five Mortality Rate (U5MR) by 2015. Measles immunization coverage is one of the indicators for progress towards MDG-4. In 2008, there were an estimated 164,000 measles deaths globally. WHO estimated that during 2000 to 2007, measles caused deaths declined by 89% in Africa. However, measles outbreaks continue to occur throughout the region [5].

In 1980, the Government of Ethiopia (GoE) initiated the implementation of EPI with goal of increasing vaccination coverage against the six childhood killer diseases by 10% each year to reach 100% coverage in 1990. This program goal has largely remained unrealized even using different efforts [6].

Despite the high prevalence of VPDs in the country, immunization coverage rates stagnated and remained very low for many years. Health Sector Development Program IV (HSDP-IV) goal of the ministry of health EPI strategy is to achieve 96% DPT-3 coverage in all regions. The major hindering factors from achieving universal immunization include: low access to services, low number of trained manpower, high staff turnover, lack of fund donors, lack of information, lack of transportation, distance from health facilities, inadequate awareness of mothers/caregivers, others such as missed opportunities, and high dropout rates especially through routine approaches [7].

The Ethiopia Demographic Health Survey 2011 (EDHS-2011) showed coverage level for DPT-3 and the percentage of fully immunized children are reportedly 36.5% and 24.3% respectively. In Oromia region DPT-3 and full immunization coverage were 26.8% and 15.6% respectively. According to EDHS-2011, DPT-3 coverage in many of the regions was below 80%, the lowest being in Afar region 9%, the highest in Tigray 73.4% and in Oromia 26.8% [8].

Infant Mortality Rate (IMR) stood at 59 per 1000 live births nationally, 73 per 1000 live births in Oromia region, and U5MR was 88 per 1000 live births for national and 112 per 1000 live births for Oromia region respectively. Reducing U5MR to 67/1000 by 2015 can only be achieved if cost effective and high impacts interventions developed in support of the child health program are implemented at very high levels of coverage which includes among others: RED strategy, Integrated Management of Childhood Illness (IMCI) and Enhanced Outreach Strategy (EOS) [9]. In connection to this, pentavalent was introduced in 2006 with objective of increasing pentavalent three coverage to 88% by the end of 2011 [10,11].

In order to increase child immunization coverage, the underlying causes and parents’ reasons not to immunize their children should be known. In the study area, so far no community based immunization coverage assessment study was conducted. Therefore, this study will try to fill these gaps by identifying the child immunization coverage and factors associated with full immunization. It will also help policy makers, program implementers and service providers to eliminate the obstacles and improve child immunization coverage in order to attain the intended prevention and control of VPDs. It also helps as a baseline for future studies.

## Methods

### Study area and period

The study was conducted in Sinana District, Bale zone, Oromia Region from December 2012 to January 2013. The district is located at a distance of 450 kilometers to southeast of Addis Ababa. Sinana district has 2 urban and 19 rural kebeles. According to the 2007 national census, the total population of Sinana district was 136,194 of which 66,735(48.99%) and 69,459(51.01%) were females and males respectively [12]; with 3,024(2.22%) 12 to 23 month old children. There were 6 health centers, 20 health posts and 14 low level private clinics and 7 rural drug vendors in the district. But, EPI was all provided by governmental health centers and health posts. According to 2011/12 district health report, 85% of children were fully vaccinated [13].

### Study design

A community based cross-sectional study design involving both quantitative and qualitative (focus group discussion and in-depth interview) approaches was employed.

### Source population

All children aged 12 to 23 months with their mothers or caregivers living in Sinana district.

### Study population

Randomly selected children aged 12 to 23 months with their mothers or caregivers living in Sinana district.

### Sample size

#### For quantitative data

The sample size required for quantitative survey was computed using a formula of calculating single population proportions with the assumption of 5% margin of error, 95% confidence level. Sample size was calculated by considering the estimated proportion of mothers knowledgeable on immunization (67.5%) which was taken from recent study in Ethiopia [14]. Sample size was calculated for both specific objectives. The estimate which yielded the highest number was considered as a final sample size. After adjustment by using finite population correction formula and adding 10% non-response rate and by considering design effect of two, the final sample size was 606 children and their corresponding parents or caregivers.

### For qualitative data

Totally, three Focus Group Discussions (FGDs) were conducted. In-depth interviews were conducted with health care providers of Primary Health Care (PHC) that included head of the selected health care provider working on immunization and Health Extension Workers (HEWs) from surrounding sub-sample of health facilities with 12 participants. Twelve reproductive age group mothers per FGD who had child 12 to 23 months of age were selected purposively from selected zones (those who are not participated in the quantitative study) during data collection. These were categorized in to three separate FGD sessions with consideration of homogeneity with respect to educational status, number of children and etc.

### Sampling technique

Initially the total kebeles (21 kebeles in the district) were stratified into rural (19 kebeles) and urban (2 kebeles) areas. Then, five rural and one urban kebeles were selected by lottery method from the total kebeles in the district. Then, from each sampled kebeles, zones or sub-kebeles were selected by lottery method. The modified 2005 WHO-EPI cluster sampling method was employed to select study households with consideration of each zones/sub-kebeles as one cluster [15].

Then, the selection of the required number of children was from the selected households with proportional allocation of study subjects. The first child in each zone was selected randomly from the center of the zone and the rest of them were selected from the subsequent household till the required number of children have been attained. For households with more than one eligible child only one child was included by lottery method.

### Data collection

It is an interviewer-administered structured questionnaire to obtain information from mothers or caregivers of the child by trained interviewers. The instrument was constructed from a review of available literature on immunization coverage, WHO questionnaire, and EDHS for immunization coverage and translated in to local language [8,15,16].

The knowledge and attitude of the mothers or caregivers was assessed by six and five questions respectively. For data collection, the interviewers used a manual prepared by the investigators to help them understand the questionnaire to collect data. For qualitative parts, FGD guides were prepared by principal investigators.

Nine data collectors and three supervisors were recruited based on a set of criteria such as knowledge of the local language Afan Oromo and previous experience on data collection. They were trained for two days by the principal investigators on the purpose of the study, data collection tools or instruments, how to consent, how to select children from households, how to interview and how to extract information from immunization card and the overall data collection procedures.

Mothers or caregivers were asked to show immunization cards and the dates of immunization were extracted from the cards. For those whose immunization cards were not available or lost, the mothers or caregivers were asked on immunization status of their children. For instance, in the case of DPT and polio, the mothers or caregivers were asked to report the number of vaccines that the child has received. In order to reduce recall bias different recalling techniques such as routes of administration (checking injection sites and presence of the scar on the upper arm, whether the child has taken vaccine orally) were used.

### For qualitative data

In-depth interviews were conducted with health professionals (probed on missed opportunities, health information delivery system and any difficulty or barriers to provide immunization service) and three FGDs were conducted with mothers or caregivers (probed on their knowledge, attitude towards immunization and barriers for not vaccinating their children). In-depth interviews were conducted by principal investigators. FGDs with mothers’ or caregivers’ were moderated by female diploma nurse. A single FGD lasts for an hour.

### Data analysis and quality management

#### For quantitative data

Data obtained from the questionnaire was entered, cleaned and prepared for tabulation using statistical data analysis software (Epi-Info version 3.5.3 and SPSS Version 20). Frequencies and other descriptive statistics were done. Bivariate analysis was conducted to examine association between dependent and independent variables; Odds Ratios (ORs) and their 95% CIs were calculated. Then, all variables that has p-value less than 0.2 in the bivariate analysis were included in the multiple logistic regression analysis model to determine the factors associated with full immunization coverage among children aged 12 to 23 months old. Adjusted Odds Ratio (AOR) with their 95% CIs were computed to determine the true association.

Data were categorized in to four groups (these groups were: socio-demographic characteristics, maternal health care use, child characteristics and mothers’ knowledge of vaccine and vaccine preventable diseases) to see the association of explanatory variables with outcome variable. Then, from each of the group variables that had p-value of less than 0.05 were entered in to final model to control for confounders and to determine true association. Qualitative (FGDs and IDIs) data were transcribed, translated to English by replaying the tape recorder and analyzed by using thematic approach (by organizing the topics raised at the time of group discussion independently).

The questionnaire was pre-tested (on 5% mothers or caregivers of children whose age was between 12 to 23 months) in kebeles which are not primarily selected for the study and the findings were excluded from main study. The necessary amendments were made upon identification of ambiguities of the tools in the wording, logic and skipping order. The principal investigators and the supervisors checked the collected data for completeness and 15(2.4%) questionnaires were rejected due to inconsistencies and incompleteness.

### Measurements

**Fully vaccinated**: a child between 12 to 23 months who received one dose of Bacille Calmette Guerin (BCG), at least three doses of pentavalent, three doses of OPV and one dose of measles vaccine by card plus mother history.

**Partially/incompletely immunized**: a child 12 to 23 months who had missed one of the eight vaccines.

**Not immunized**: a child 12 to 23 months who didn’t receive any vaccine before the study.

**Coverage by card only**: coverage calculated with numerator based only on documented dose, excluding from the numerator those vaccinated by history.

**Coverage by card plus history**: coverage calculated with numerator based on card and mother’s report.

**Missed opportunity**: eligible child for vaccination had gone to health facility, but didn’t receive vaccine for which he or she is eligible at that day.

**Sufficient knowledge**: six knowledge questions were asked and correct answers were given a score one and incorrect answers scored zero. Those scores which are greater than the mean were classified as having sufficient knowledge.

**Positive attitude towards immunization**: when the respondents positively reacted to at least three out of the four attitude questions regarding immunization.

**Caregiver**: is the most responsible person that provides child care for the 12 to 23 months old child whose biological mother couldn’t provide the intimate care.

**Index child**: refers to 12 to 23 months child that is included randomly in the study from a household. In case, when more than one eligible child is present in a given household, one of them was selected at random to be included in the study.

**Literate**: mothers or caretakers or fathers with formal education or able to read and write.

**Dropout rate (DOR)**: is the rate difference between the initial vaccines (BCG or Pentavalent I) and the final vaccines (Pentavalent III or Measles).

**BCG to Measles dropout rate**: the percent of children vaccinated for BCG who doesn’t receive measles vaccine.

$$ \mathrm{B}\mathrm{C}\mathrm{G}/\mathrm{Measles}\ \mathrm{dropout}\ \mathrm{rate}\ \left(\mathrm{over}\ \mathrm{all}\ \mathrm{dropout}\ \mathrm{rate}\right) = \frac{\left(\mathrm{B}\mathrm{C}\mathrm{G} - \mathrm{Measles}\right)}{\mathrm{BCG}}*\ 100\% $$

**Pentavalent I to pentavalent III dropout rate**: the percent of children vaccinated for pentavalent I, but who did not receive pentavalent III.

$$ \mathrm{Penta}\mathrm{valent}\ \mathrm{I}/\mathrm{Pentavalent}\ \mathrm{I}\mathrm{I}\mathrm{I}\ \mathrm{dropout}\ \mathrm{rate} = \frac{\left(\mathrm{Penta}\ \mathrm{I} - \mathrm{Penta}\ \mathrm{I}\mathrm{I}\mathrm{I}\right)}{\mathrm{Penta}\ \mathrm{I}}*\ 100\% $$

### Ethical considerations

The research was approved by the research ethics review committee of the School of Public Health of Addis Ababa University before conducting the study. Permission to undertake the study was obtained from every relevant authorities in the zone, district and respective kebeles. Applicable consent form and the information sheet were duly integrated along with the respective data collection instruments. All the study participants were clearly informed about the objectives or purposes, procedures, risks and benefits, privacy and confidentiality issues of the study. Finally, verbal informed consent was obtained from each study participant before interview. This method of consent was specifically approved by the ethical committee that approved our study.

## Results

### Socio-demographic characteristics of mothers or caregivers

A total of 591 mothers or caregivers of children aged between 12 to 23 months were interviewed with a response rate of 98.5%. Of the total 591 respondents, 562(95.1%) were mothers of the children and 29(4.9%) were caregivers. Of the 591, 478(80.9%) and 113(19.1%) of them were rural and urban residents respectively. The majority 575(97.3%) of respondents belong to Oromo ethnic group, 313(53%) of them Orthodox Christian followed by 265(44.8%) Islam in religion.

The median age of the mothers or caregivers was 28(±6.1 SD) years, which ranges from 17 to 58 years. From the total respondents, 340(57.5%) attended primary school, while 60(10.2%) completed secondary school and above level and 191(32.3%) of them can’t read and write. In line to this, 309(52.3%), 110(18.6%) and 172(29.1%) of the fathers (head of households) have attended primary school, secondary school or above and they have not gone to school for formal education respectively. More than half, 326(55.1%) of mothers or caregivers and 528(89.3%) of fathers were farmers. Almost three fourth of the families, 432(73.1%) possess radio and only 141(23.9%) own television. Their mean monthly household income was 763.4 Ethiopian Birr (ETB) or ~38 USD (±725.98 SD) and varying from 100 to 5000 ETB or ~5 to 250 USD.

### Socio-demographic characteristics of children

From a total of 591 children included in the study, 239(40.4%) were females, 352(59.6%) were males and 175(29.6%) of them aged exactly 23 months. The overall mean age of the children was 17.9(±4.2 SD) months. From the total children who have participated in this study, 576(97.5%) were vaccinated at least once and 15(2.5%) were never vaccinated. Of those who have already vaccinated, 338(58.7%) and 218(37.8%) of them started the vaccine in age of ≤ 1 month and between 2 and 3 months respectively. Parents showed vaccination card almost for one third, 195(33%) of the children during the survey (refer Table 1).

**Table 1:** **Socio-demographic characteristics of children aged 12 to 23 months in Sinana district, Bale zone, Oromia region, Southeast Ethiopia, 2012/13**

**Variables**		**Frequency**	**Percent (%)**
Child’s place of delivery			
	Health facilities	190	32.1
	Home	401	67.9
Child’s birth order			
	First	103	17.4
	Second	125	21.2
	Third	132	22.3
	Fourth	82	13.9
	Fifth and above	149	25.2
Child ever vaccinated			
	Yes	576	97.5
	No	15	2.5
Age at which child started vaccination (in months)			
	≤1	338	58.7
	2 to 3	218	37.8
	4 and above	20	3.5
Children having vaccination card			
	Yes	190	33.0
	No	386	67.0

### Availability and accessibility of vaccination services

Almost all, 584(98.8%) of respondents reported that they have access to health facilities that provide immunization services. Majority of them, 537(92.0%) reported that they have more access to health post, 382(76.9%) have access to services provided during outreach and 270(46.2%) have access to health center. As far as average distance to health facility in travel hours or minutes was concerned, 289(49.5%) of respondents have travelled ≤ 15 minutes and 10(1.7%) travelled greater than an hour (refer Table 2).

**Table 2:** **Availability and accessibility of the vaccination services in Sinana district, Bale zone, Oromia region, Southeast Ethiopia, 2012/13**

**Variables**		**Frequency**	**Percent (%)**
Presence of health facility			
	Yes	584	98.8
	No	7	1.2
Number of health facility			
	Health Center	270	46.2
	Health Post	537	92.0
Presence of outreach site			
	Yes	497	85.1
	No	87	14.9
Number of active outreach site (in number)		382	76.9
Average distance to health facility in travel hours or minutes			
	≤15 minutes	289	49.5
	>15 minutes to <30 minutes	173	29.6
	≥30 minutes to ≤ 1 hour	112	19.2
	>1 hour	10	1.7
Convenience of services on vaccination			
	Yes	570	98.9
	No	6	1.1
Presence of HEWs in the kebeles			
	Yes	585	99.0
	No	6	1.0
HEWs visited your home			
	Yes	573	97.9
	No	12	2.1
Given information on vaccination by HEWs			
	Yes	564	96.4
	No	21	3.6

From the total, 573(97.9%) of the households were visited by HEWs and 564(96.4%) were given information on immunization by HEWs regularly. Of the total 576 mothers visited health facilities for vaccination, 119(20.7%) turned back home without getting their children vaccinated. From this, the 69(58.0%) were due to the unavailability of the service providers and 62(52.1%) were due to lack of vaccine at these facilities.

### Knowledge and attitudes of mothers/caregivers on vaccination and VPDs

Concerning knowledge of mothers on vaccination and VPDs; almost all, 573(97.0%) have ever heard about vaccination. Majority of them, 548(95.6%) heard about vaccination from HEWs and 526(91.8%) heard from media (radio, television, newspapers etc.).

Almost all, 579(98.0%) have replied that immunization prevents communicable diseases and 494(83.6%) of them knew VPDs. As far as age at which children will receive BCG vaccine is concerned, 193(43.8%) said at birth, 226(51.2%) at 2 weeks after birth and 21(4.8%) at six weeks for BCG vaccine. Whereas for measles, 362(77.7%) reported at six months, 102(21.9%) at nine months and 2(0.4%) at twelve months. Out of the 518 who knew about when the child should complete the immunization, 509(98.3%) reported the completion of immunization (i.e. before a year).

Study subjects were also asked for symptoms of VPDs and majority of them, 460(98.1%) reported rash of measles, 452(96.4%) cough and 446(95.1%) paralysis in case of polio. Overall more than two-third, 421(71.2%) of the study subjects were knowledgeable (have good knowledge and scored above the mean i.e. 4.95(±1.5 SD); whereas 170(28.8%) were completely non-knowledgeable (poor knowledge) regarding immunization. The summarized attitudinal index indicates that 587(99.3%) of the total respondents have favorable attitude towards immunization services utilization; while the remaining 170(28.8%) have unfavorable attitude.

### Immunization coverage by card plus mother recall

Based on the vaccination card and the mothers’ recall, almost all 576(97.5%) of the children took at least a single dose of vaccine. From the total vaccination report, 454(76.8%) were immunized completely (95% CI: 73% to 80%). Of the recommended vaccine doses in general, polio is the most frequently taken vaccine. Particularly OPV-1 was reportedly taken by 97.0% of the children followed by 95.9% of Penta-1, 93.6% of OPV-2, 93.2% of Penta-2 and 85.4% received OPV-3. Measles (77.7%) was the least received vaccine.

More than three-fourth, (84.6%) of the children have taken Penta-3 with 11.8% pentavalent dropout rate, 19.5% Penta-1 to measles dropout rate and 15.8% overall dropout (BCG to measles) rates. The coverage of immunization showed decrement from the initial doses of vaccine to the last doses. Based on the information extracted from vaccination card, only 152(25.7%) children completed all the recommended doses of vaccines.

### Factor affecting immunization completion for children

In this study, factors associated with full immunization coverage were assessed. These factors include socio-demographic characteristics of mothers or caregivers and children, maternal health care utilization, availability and accessibility of health care services, knowledge of mothers or caregivers on vaccination and VPDs.

### Socio-demographic characteristics of mothers/caregivers

Results from bivariate analysis indicated that maternal education, maternal occupation, father’s education, family income and presence of radio or television in the house were the factors that were significantly associated with the increased completion of immunization among 12 to 23 months of children.

The presence of television and radio in their home also showed difference in completion of child immunization in multiple logistic regression. Children from the family who had television were 1.6 times more likely to complete their immunization than family who had no television (AOR = 1.6; 95% CI: 1.03, 2.66). But, marital status, religion, ethnic group, occupation of father, family size and presence of radio in the house did not show an association on completion of child immunization in multiple logistic regression.

After adjusting for the other variables, only occupation of mother, educational level of father and family income remained significant in in multiple logistic regression. Concerning occupation, taking housewives as reference, children whose mothers are farmers were 1.9 times more likely to be fully vaccinated (AOR = 1.9; 95% CI: 1.1, 3.1).

Children whose fathers have attended primary and secondary schools were 1.9 and 3.1 times more likely to be fully vaccinated than whose father unable to read and write [(AOR = 1.8; 95% CI: 1.02, 3.11) and (AOR = 3.1; 95% CI: 1.3, 7.4)] respectively. Concerning average monthly income of the family, children from the household with average monthly income >1000 ETB were 3.2 times more likely to complete their vaccination than children from the household with average monthly income of < 500 ETB (AOR = 3.2; 95% CI: 1.4, 7.4).

### Maternal health care utilization

Antenatal care (ANC) follow up, post-natal care (PNC) follow up, whether mothers/caregivers ever visited health institutions and visited health facility specifically for immunization were assessed in bivariate analysis and all factors showed association with completion of child immunization (refer Table 3 for the descriptive and refer Table 4 for associated factors)**.**

**Table 3:** **Maternal health care utilization in Sinana district, Bale zone, Oromia region, Southeast Ethiopia, 2012/13**

**Variables**		**Frequency**	**Percent (%)**
ANC visit			
	Yes	435	73.6
	No	156	26.4
PNC visit			
	Yes	305	51.6
	No	286	48.4
Ever visit health facility for any purpose with child			
	Yes	572	96.8
	No	19	3.2
Child received vaccines that day of visit			
	Yes	568	99.3
	No	4	0.7

**Table 4:** **Completion of immunization among children aged 12 to 23 months by maternal health care utilization, Sinana district, Bale zone, Oromia region, Southeast Ethiopia, 2012/13**

**Variable**		**Fully vaccinated**		**Odds ratio (95% CI)**	
		**Yes**	**No**	**Crude**	**Adjusted**
ANC visit					
	Yes	359 (60.7)	76 (12.9)	3.0 (2.0, 4.6)*	3.7 (2.3, 5.9)*
	No	95 (16.1)	61 (10.3)	1.00	1.00
PNC visit					
	Yes	250 (42.3)	55 (9.3)	1.8 (1.2, 2.7)*	1.0 (0.6, 1.8)
	No	204 (34.5)	82 (13.9)	1.00	1.00
Visit health facility for any purpose					
	Yes	452 (76.5)	120 (20.3)	32 (7.3, 140.5)*	NI
	No	2 (0.3)	17 (2.9)	1.00	
Child receive vaccine that day					
	Yes	450 (78.4)	120 (20.9)	3.8 (0.5, 26.9)	1.8 (0.1, 21.7)
	No	2 (0.3)	2 (0.3)	1.00	1.00

N.B: numbers in brackets are in percentage, NI- Variable not included in the model.

*Significant at P-value of <0.05.

After adjusting for the other variables, only ANC utilization remained statistically significant in multivariate logistic regression. Mothers who have utilized ANC during the pregnancy of the index child were 3.7 [(95% CI: 2.3, 5.9)] times more likely to fully immunize their children than mothers who have not utilized.

This finding is supported by the FGDs conducted with mothers. All discussants believed that visiting health facility during pregnancy and after delivery are very crucial for mothers and their children. The reasons forwarded were: if mother went to the health facility during pregnancy and post-delivery; health professionals would give advice and information on the progression of pregnancy, well-being of baby, alternative place of delivery and what to do after giving birth. Mothers could also be counselled on child immunization and how to feed the new born baby.

A 20 years old rural woman said that: *“… when a mother took her child to health facility (to the health professionals), it is a good opportunity for getting advice and guidance on the initiation time of vaccines, when it should get completed and the importance of completing immunization for children. So, having ANC and PNC follow up is necessary for all mothers and their children…”*

### Availability and accessibility of health care services

The association of health care availability and accessibility with the completion of vaccination were also seen by using bivariate and multivariate analysis (refer Table 5). Despite bivariate level significance, only average distance to the health facility in travel hours or minutes showed significant association in multivariate analysis, and mothers or caregivers who have travelled less than an hour were 3 times more likely to get their children fully vaccinated than mothers or caregivers travelled less than 15 minutes (AOR = 3.1; 95% CI: 1.5, 6.3).

**Table 5:** **Completion of Child immunization among 12 to 23 months by availability and accessibility of health care service, Sinana district, Bale zone, Oromia region, Southeast Ethiopia, 2012/13**

**Variable**		**Fully vaccinated**		**Odds ratio (95% CI)**	
		**Yes**	**No**	**Crude**	**Adjusted**
Average distance to health facility in travel hours or minutes					
	≤15 minutes	210 (35.5)	79 (13.4)	1.00	
	>15 minutes to <30 minutes	136 (23)	37 (6.3)	1.4 (0.9, 2.2)	1.6 (0.9, 2.6)
	≥30 minutes to ≤ 1 hour	100 (16.9)	16 (2.7)	2.4 (1.3, 4.2)*	3.1 (1.5, 6.3)*
	>1 hour	8 (1.4)	5 (0.8)	0.6 (0.2, 1.9)	0.8 (0.2, 2.9)
Service convenient					
	Yes	448 (77.5)	122 (21.1)	6.0 (1.4, 25.9)*	NI
	No	3 (0.5)	5 (0.9)	1.00	
Long waiting time at facility					
	Yes	420 (73.7)	117 (20.5)	0.6 (0.2, 1.7)	1.7 (0.6, 4.6)
	No	28 (4.9)	5 (0.9)	1.00	
Turned back home without getting vaccine					
	Yes	91 (15.4)	28 (4.7)	0.9 (0.6, 1.6)	0.8 (0.5, 1.3)
	No	363 (61.4)	109 (18.5)	1.00	
Presence of HEWs in kebeles					
	Yes	449 (76)	136 (23)	0.7 (0.1, 5.7)	NI
	No	5 (0.8)	1 (0.2)	1.00	
HEWs given information					
	Yes	437 (74.7)	127 (21.7)	2.6 (1.1, 6.3)*	0.5 (0.2, 1.6)
	No	12 (2.1)	9 (1.5)	1.00	

N.B: numbers in brackets are in percentage, NI- Variable not included in the model.

*Significant at P-value of <0.05.

### Knowledge of mothers or caregivers on vaccination and VPDs

Associations of mothers’ knowledge about vaccination and VPDs with the completion of the Child immunization were third factor assessed (refer Table 6). After adjusting for the other variables, only two variables remained significant in multivariate logistic regression. Children whose mothers had good knowledge on vaccine and VPDs were 2.5 times more likely to be fully vaccinated than children of mothers who had poor knowledge on vaccine and VPDs [AOR = 2.5; 95% CI: 1.5, 4.2)]. Similarly, mothers who ever have discussed on vaccination with HEWs were 2.4 times more likely to complete the immunization of their children than mothers who have not discussed on immunization with HEWs [(AOR = 2.4; 95% CI: 1.3, 4.2)].

**Table 6:** **Completion of immunization among children aged 12 to 23 months by mothers or caregivers knowledge on vaccine and VPDs in Sinana district, Bale zone, Oromia region, Southeast Ethiopia, 2012/13**

**Variable**		**Fully vaccinated**		**Odds ratio (95% CI)**	
		**Yes**	**No**	**Crude**	**Adjusted**
Ever discussed on immunization with HEWs					
	Yes	372 (62.9)	74 (12.5)	3.9 (2.6, 5.8)*	2.4 (1.3, 4.2)*
	No	82 (13.9)	63 (10.7)	1.00	
Ever encouraged to immunize last year					
	Yes	422 (71.4)	103 (17.4)	4.4 (2.6, 7.4)*	4.9 (1.0, 21.3)*
	No	32 (5.4)	34 (5.8)	1.00	
Attitude of mothers/caretakers toward vaccination					
	Negative attitude	3 (0.5)	1 (0.2)	1.00	
	Positive attitude	451 (76.3)	136 (23)	1.11 (0.1, 10.7)	NI
Knowledge of mothers/caretakers on vaccination					
	Poor knowledge	112 (18.9)	58 (9.8)	1.00	
	Good knowledge	342 (57.9)	79 (13.4)	2.2 (1.5, 3.4)	2.5 (1.5, 4.2)*

N.B: numbers in brackets are in percentage, NI- Variable not included in the model.

*Significant at P-value of <0.05.

Findings of the FGDs also indicated that majority of mothers or caregivers remember immunization day during announcement for vaccination campaign. As discussants indicated, announcement at outreach site for vaccination of children is held each month on weekends. So, this is convenient for mothers to get their children vaccinated.

A 34 years old uneducated mother from rural said that: *“… I use outreach service to vaccinate my children. I remember the date from the announcement and since the outreach site is not too far, I’m vaccinating my children regularly by using such advantage. If other health problems happened on my children I took them to health facility, however in most of the cases for utilization immunization services, I use outreach site.”*

### Child characteristics

The association of the child characteristics like sex, place of delivery and birth order with completion of child immunization were the variables assessed by this study. From these variables, only child birth order showed significant association with completion of immunization in both bivariate and multivariate logistic regression analysis. The likelihood of immunization completion among mothers with first birth order was less by 30% as compared with mothers with third birth order (AOR: 0.3; 95% CI = 0.2, 0.4) (refer Table 7).

**Table 7:** **Immunization completion among children aged 12 to 23 months by child characteristics in Sinana district, Bale zone, Oromia, Southeast Ethiopia, 2012/13**

**Variable**		**Fully vaccinated**		**Odds ratio (95% CI)**	
		**Yes (%)**	**No (%)**	**Crude**	**Adjusted**
Sex of a child					
	Female	275 (46.5)	77 (13.0)	1.00	
	Male	179 (30.3)	60 (10.2)	1.2 (0.8, 1.8)	NI
Child place of delivery					
	Home	307 (51.9)	94 (15.9)	1.00	
	Health facility	147 (24.9)	43 (7.3)	1.1 (0.7, 1.6)	NI
Child birth order					
	First	86 (14.6)	17 (2.9)	1.00	
	Second	98 (16.6)	27 (4.6)	0.7 (0.4, 1.4)	1.6 (0.7, 3.6)
	Third	95 (16.1)	37 (6.3)	0.5 (0.3, 0.9)*	0.3 (0.2, 0.4)*
	Fourth	57 (9.6)	25 (4.2)	0.5 (0.2, 0.9)*	0.7 (0.4, 1.4)
	Fifth	118 (19.9)	31 (5.2)	0.8 (0.4, 1.5)	0.6 (0.3, 1.2)

N.B: NI- Variable not included in the model.

*Significant at P-value of <0.05.

**Table 8:** **Multivariate analysis for completion of child immunization (full immunization) and selected variables in Sinana district, Bale zone, Oromia region, Southeast Ethiopia, 2012/13**

**Variables**	**Fully vaccinated**		**Odds ratio (95% CI)**	
	**Yes**	**No**	**Crude**	**Adjusted**
**Occupation of the mothers or caregivers**				
Housewife	156	69	1.00	1.00
Farmer	266	60	1.9(1.3, 2.9)	1.7(1.0, 2.8)*
Others	32	8	1.8(0.8, 4.0)	0.6(0.2, 1.9)
**Educational level of father**				
Unable to read and write	117	55	1.00	1.00
Primary level	240	69	1.6(1.1, 2.5)	1.6(0.9, 2.6)
Secondary and above level	97	13	3.5(1.8, 6.8)	2.8(1.3, 6.2)*
**Family income in ETB**				
100 to 500	237	89	1.00	1.00
501 to 1000	142	35	1.5(0.9, 2.4)	1.2(0.7, 2.0)
>1000	75	13	2.2(1.2, 4.1)	3.0(1.3, 6.9)*
**Visit ANC**				
Yes	359	76	3.0(2.0, 4.6)	3.8(2.4, 6.4)*
No	95	61	1.00	1.00
**Average distance to health facility in travel hours or minutes**				
≤15 minutes	210	79	1.00	1.00
>15 minutes to <30 minutes	136	37	1.4(0.9, 2.2)	1.5(0.9, 2.5)
≥30 minutes to ≤ 1 hour	100	16	2.4(1.3, 4.2)	3.0(1.5, 6.1)*
>1 hour	8	5	0.6(0.2, 1.9)	0.7(0.2, 2.6)
**Ever discussed on immunization with HEWs**				
Yes	372	74	3.9(2.6, 5.8)	2.1(1.2, 3.9)*
No	82	63	1.00	1.00
**Knowledge of mother on immunization**				
Poor knowledge	112	58	1.00	1.00
Good knowledge	342	79	2.2(1.5, 3.4)	2.3(1.3, 3.9)*

*Significant at P-value of <0.05.

### Reasons for not being vaccinated among partially/unvaccinated children

From the mothers/caregivers not immunized or not completed immunization for their children 85(62.0%) responded due to lack of awareness on the necessity to return for second and third doses of vaccine, 47.4% reported place or time of immunization is not known and 14.6% of the mothers said that place of immunization is too far from home.

On the other hand, fear of adverse reaction (55.5%), wrong ideas about contra-indications (48.2%), lack of trust on immunization (38.7%) and no confirmed information on immunization were reasons for not immunizing. In relation to this, FGDs indicated lack of awareness, fear of side effect, mothers give less attention to child immunization and males’ involvement in child immunization is very low.

*“… A friend of mine living in the rural has five children. She didn’t gave birth at health facilities and none of her children were vaccinated because her husband didn’t allow her to go health facilities for child immunization because she is burdened with many activities at home…”* said by 25 years educated urban mother.

Other idea raised was fear of adverse reaction or side effects of the vaccinations which might discourage mothers not to go health facilities for the second visit.

Thirty years old rural woman said that: *“… most of the children automatically develop fever and unusual discomfort for at least three days after getting vaccination which is really frustrating situation till they recovered even for families. I fear vaccination might decease my child. So, I never go again to vaccinate my child because I have seen the episode…”*

Health workers also indicated that negative attitude of the mothers or caregivers toward immunization; lack of trust on immunization are among the chief reasons that contributed a lot for low coverage of immunization in the district. Moreover, the mothers or caregivers didn’t fully understood the benefits of vaccination rather they considered as it brought about fever and other discomforts to the children.

Thirty four years old mother stated that: *“… mothers or caregivers lack awareness on benefits of vaccines. This might lead them to believe that vaccines cause diseases. Sometimes when observed practically after getting vaccination children will develop high fever and significant discomforts and then mothers or caregivers never return to the subsequent doses or appointment.”*

On the other hand, health workers agree that health information dissemination sessions which were given in mass do have problems. Mothers’ or caregivers’ level of understanding and educational background were not well recognized in particular. So, this could not motivate mothers for return.

*“…on providing quality health information, we do have problems. It is not fully understood by our clients. It doesn’t meet the expectations of the communities (specifically rural communities) in conveying the right kind information regarding immunization in casting out misconceptions. Yet, information we have provided, didn’t bring the desired behavioral change…,”* said by 32 years old male health professional.

## Discussion

Immunization is one of the most successful and cost-effective public health interventions. Providing immunization service also offers an opportunity to deliver other preventive services, like vitamin A supplements and deworming. But, parents still do not perceive immunization as a right, and demand for immunization service is lacking or low in many developing countries. Antenatal care follow up, educational level of fathers, occupation of mothers and family income were some of the factors significantly associated with immunization coverage in this study. Despite the improvements, immunization is unfinished agenda in many developing countries including Ethiopia. So, this study tried to assess the full immunization coverage and factors associated with it among 12 to 23 months old children residing in Sinana district.

Oral polio vaccine coverage was slightly higher than the coverage of the pentavalent vaccine which is given in line with EPI schedule of Ethiopia. This is probably due to the OPV vaccine is given frequently as national campaign in the country. On the other hand, Penta-3 vaccine coverage was a bit higher than measles vaccine coverage which could be as a result of time gap between two vaccines in which mothers may forget the measles vaccine and dropout from the consequent doses.

Across all vaccine doses, from first to consequent doses, there is decrement of coverage which could be due to mothers or caregivers incompliance and time gap between each dose leading mothers to forget the subsequent doses. So, the dropout rate of Penta-1 to Penta-3 was 11.8%, Penta-1 to measles 19.5% and overall BCG to measles dropout was 15.8%. The above figures are higher than the international goal set by WHO (i.e. making <10%). But, this finding is less than the study done in Oromia region in which Penta-1 to Penta-3 dropout rate was 33% [17].

When we compare immunization coverage of Sinana district with that of Kafa [18], the Sinana coverage showed that 11.2% increment. This could be due to time gap between two studies and awareness of mothers on immunization could be changed over time. Availability and accessibility of the services might be other reason making these differences.

Similarly, when we compare immunization coverage of Sinana district with EDHS 2011, the percent of fully vaccinated is higher and proportion of children unvaccinated were decreased by 12.5%. This is likely due to EDHS 2011 report included data from areas of low immunization coverage and time of the survey could also be another reason for the discrepancy. But, coverage of Penta-3, measles and fully immunized in Sinana district is lower than the immunization coverage reported in 2011 national and Oromia region health and health related indicators [8,11]. The percentage of fully vaccinated reported in this study (76.8%) was also lower than the district health office report of 2011, which was 85%. This is probably due to methods used, sample size, areas covered, over reporting and type of data sources used [13].

Among interviewed mothers, only 190(33%) of the children immunization card was confirmed. Most of the children took OPV-1, followed by Penta-1. But, measles vaccine was the least utilized vaccine and 76.8% (95% CI: 73%, 80%) of children completed the recommended doses of immunization.

Almost all, 97% of mothers have heard about immunization. More than three fourth (83.6%) of mothers knew VPDs. Majority of mothers heard on radio (91.8%), which might indicate that mothers had access to medias. Kebeles in which village leaders participated in EPI program by giving information to habitants, motivating mothers and community on health service utilization also showed improvement in immunizing their children. This in turn might reveal the importance of political commitment to improve immunization status of children.

From this, more than half of the respondents knew at least more than six VPDs which are higher than study carried out in Ambo district in which majority of mothers knew more than three VPDs [14]. This difference might indicate that the information about immunization is made available for potential users in the study area.

In this study, mothers or caregivers educational status is among determinants of immunization completion and those mothers or caregivers who attended secondary and above level were two times more likely to complete the immunization of their children than mothers unable to read and write. This is may be as educational status of family gets improved, health seeking behavior of family may perhaps increase. This in turn may have positive impact on child immunization.

Other factor showed association with child immunization completion were fathers’ educational level. This is consistent with finding from EDHS 2011, Istanbul, Northern Nigeria, Burkina Faso [8,16,19,20]. This could be due to household decision making power of father and awareness of father on vaccine and VPDs might make fathers at good position to vaccinate their children.

In this study, sex of child and place of delivery were not showed significant association with vaccine completion among 12 to 23 months old children. But, study from other places indicated that these factors have a significant association with child immunization status [8,14,22]. However, this study is consistent with survey in Mozambique in which gender has no difference in completing vaccination [23].

Mothers whose occupation is farmer were 1.7 times more likely to complete the immunization of child than housewives; the proportion of not fully vaccinated children are higher among housewives. This is similar with study in Jimma town, south west Ethiopia [24].

Family income is other factor included in multivariate; children from household whose average monthly income is greater than 1000 ETB were three times more likely to be fully vaccinated than whose income is low. If income of family is high, they will have access to medias, probably exposed to information through these media. This finding is consistent with study done in different areas [8,20,25].

Maternal health care utilization was associated with child immunization completion among 12 to 23 months; children whose mothers had ANC follow up were more likely to be fully vaccinated than who did not attend ANC. This finding is consistent with that of India, Ambo, Mali, Nigeria [14,22,26,27]. This could be due to mothers health seeking behavior and mothers may discuss with health professionals on vaccine and VPDs, on importance of immunization, time of vaccine initiation, when it could get completed and possible side effect associated to vaccine during the follow up. So, it may create good opportunity for mother to get their children vaccinated. This could also motivate mothers to use health facility services.

Average walking time is other factor showed association with completion of child immunization. This finding is inconsistent with that of Philippines in which, as distance from health facility get more than 0.5 km, the immunization coverage decreased. In addition to this, study from Mozambique showed that distance from health facility hinders immunization of children [23,28].

A 28 years old supervisor of HEWs in the district said that: “… *the presence of HEWs working in community and outreach service which held on public holidays which mobilizes community to immunization monthly might played a significant role. So, this could help mothers to easily remember immunization day to vaccinate their children…”*

A 35 years old mother from rural area said that: *“… I’m using outreach services to get my child vaccinated by getting noticed through announcement and since distance of this outreach area is not too far I prefer to use this option. I’m vaccinating my child some times when my child gets sick at health post or health center.”*

Concerning knowledge of mothers on vaccine and VPDs; children whose mothers classified as having sufficient knowledge on immunization were twice more likely to be fully vaccinated than whose mother had little knowledge. This study is consistent with study done in Oromia region Ambo district, and Nouna district, Burkina Faso, Nigeria district, case control study in Wonago district south Ethiopia [20,21,24,29], as knowledge of mother improved on immunization, they could develop positive attitude. Then, they were motivated to complete the immunization.

Child birth order is associated to child immunization completion; and child born to the third and above birth order is 40% less likely to be fully vaccinated than first birth order. That means high proportion of children were found to be fully vaccinated among first birth order which is consistent to EDHS 2011 and study from Brazil [8,21] finding in which child birth order related to vaccine completion. This could be child born to first birth order may get special focus since it has no resource competition and mothers may follow ANC for first child which may be related to health care utilization indeed.

The strength of this study is its inclusion of children aged 12 to 23 months which may measure recent immunization program performance and immunization completion rate. Information was triangulated by both quantitative and qualitative methods.

Immunization coverage by report of mother may under/over report the immunization coverage because mothers might not remember doses that child took due to recall bias. Being cross sectional study design which does not show the cause effect relationship is the other limitation of the study.

## Conclusions

More than three fourth (76.8% (95%CI: 73%, 80%)) of children were fully vaccinated. Almost all (97%) of mothers heard about immunization and 95.6% of the mothers heard from health extension workers. 98% of the mothers knew as immunization prevents communicable diseases and 71.2% of the mothers have sufficient knowledge on immunization. Among mothers participated in this study, 99.3% of them have positive attitude regarding vaccination. From the total children included in the study, only 33% of them have immunization card. Occupation of mothers/caretakers, household family income, educational level of father, sufficient knowledge, ever discuss about immunization, ANC follow up and average walking time were statistically significant predictors of fully immunization of children.

Being unaware of need for immunization, unaware of need to return for second or third dose, unknown place and/or time of immunization, fear of side effect, wrong ideas about contraindications, absence of faith in immunization, inconvenient time of immunization absence of vaccinator and vaccine and long waiting time at health facility were reasons for not fully immunizing their children.
